# Sequence Analysis of the Full-length cDNA and Protein Structure Homology Modeling of FABP2 from *Paralichthys Olivaceus*

**DOI:** 10.4137/bbi.s2287

**Published:** 2009-06-04

**Authors:** Xiaowu Chen, Zhiyi Shi

**Affiliations:** College of Fisheries and Life Science, Shanghai Ocean University, Shanghai, China

**Keywords:** paralichthys olivaceus, intestinal fatty acid-binding protein, *in silico* cloning

## Abstract

Using zebrafish intestinal fatty acid-binding protein 2 (FABP2) mRNA sequence as the initial query probe, four highly homologous *Paralichthys olivaceus* EST sequences were retrieved from Genbank database. The assembled full-length cDNA contains the open reading frame of *P. olivaceus* FABP2 gene, which was validated by subsequent RT-PCR cloning. In the coding region, the average GC content is 56%, but it would reach 76.8% if only counting for the third base of the codons. The deduced *P. olivaceus* FABP2 polypeptide contains 132 amino acids (aa), with a predicted molecular size of 15.3 kD and pI at 6.74. This protein multiple-alignment has shown that this peptide is 75.7% identical to the corresponding homologous protein in Danio rerio. Among the 7 aa that are essential for FABP2 function, 3 were found to be conserved among *P. olivaceus*, *Danio rerio*, *Tetraodon nigroviridi*, *Rattus norvegicus*, and *Homo sapiens*. The study provides essential information on molecular evolution and function of FABP family.

## Introduction

*Paralichthys olivaceus* belongs to Pleuronectiformes kingdom, *Paralichthyidae* family and *Paralichthys* genus. It is a cold water demersal fish, one of the important species for sea water fish farming. The muscles and liver cells of the fishes grown in artificial system have high a lipid content, which affects the quality of fish meat. Cloning and analysis of genes for lipid metabolism will provide basic knowledge to help understand this problem. Fatty acid-binding protein (FABP) is a key protein in lipid metabolism. FABP2 normally contains 126–137 amino acids (aa) with an apparent molecular weight between 14–16 kD. In the human body, FABP2 participates in lipid metabolism, which is regulated synergistically with other proteins including transforming growth factor beta1 (TGF-beta1), GATA binding protein 4 (GATA 4), mothers against decapentaplegic homolog 4 (SMAD 4), and glutaminyl-tRNA synthase (glutamine-hydrolyzing)-like 1 (QRSL 1).^1^ FABP2 also affects animal growth and development. FABP2 proteins are found in animal intestine, liver, heart and bone muscle, and other tissues where they are involved in fatty acid metabolism including its absorption, transportation, and regulation of the concentration gradient across cellular membranes.^2^ However, this gene has only been reported in a few species of hard-bone fishes including zebrafish (*D. rerio*), puffer fish (*T. nigroviridis*) and Asian catfish (*Pangasius hypophthalmus*). Investigating the structure, function, regulation and the interactions of fatty acid-transport proteins may provide a new way of diagnosing and treating diseases or disorders of lipid metabolism such as obesity.^3^

The *in silico* gene cloning method was developed as a result of the genome project.^4^ It uses a computer program to assemble Genbank expression sequence tags (EST) to obtain the full-length of interesting genes. This study employed this approach to clone the coding sequence for the FABP2 gene in *P. olivaceus.* Subsequent comparative and systematic analysis of the nucleotide composition, codon usage, DNA molecular evolution, protein structure and the potential mutation sites were performed to provide information for future functional study of this gene.

## Materials and Methods

### *In silico* cloning of the *P. olivaceus* FABP2 gene

One FABP2 EST clone (accession, CX285463.1) was initially identified by blast searching (Blastn program) the *P. olivaceus* EST database using zebrafish FABP2 mRNA (accession:AF180921.2) as the query sequence. Subsequently, the EST sequence was compared in the same database to retrieve the additional 3 EST sequences (accession: CX285611; CX285566; CX284708). The four EST sequences were then assembled to obtain the full-length cDNA sequence.

### Fish materials and gene validation using RT-PCR

*P. olivaceus* fishes were purchased from a local aquatic produces market. After opening the stomach, the tissues were dissected and stored in liquid nitrogen. Total RNA was isolated by guanidinium thiocyanate/cesium trifluoroacetate gradient centrifugation method. One microgram of total RNA was reverse transcribed with oligo (dT) primers and AMV reverse transcriptase in 20 μL of reaction mixture according to the manufacturer’s instructions (TAKARA, Inc., first-stand cDNA synthesis Kit). cDNAs were used to amplify the FABP2 open reading frame (ORF) utilizing a primer pair (Forward primer: ACCAGTC-GCCTTCAACCTAACACCA, and Reverse primer (CCGCTGCTGATTTTATTCACTTTCT), and following the PCR reaction conditions specified by the manufacturer (TAKARA, Inc., first-stand cDNA synthesis Kit). The PCR product was purified from 1% agarose gel and cloned onto pUCm-T vector using T4 DNA ligase. The plasmid was transformed into DH5α competent cells. The positive transformants were selected on LB plates supplemented with 5-bromo-4-chloro-3-indolyl b-D-galactopyranoside (X-gal), and isopropyl-beta-D-thiogalactopyranoside (IPTG). The cloned *P. olivaceus* FABP2 cDNA was sequenced, and deposited into Genbank (accession: EU159579).

### Bioinformatics analysis of the FABP2 cDNAs and proteins

Fourteen FABP2 cDNA sequences, each from one vertebrate species were retrieved from Genbank. The FABP2 protein sequences in human (*Homo sapiens*), rat, puffer fish and zebrafish were searched in SWISS-PROT database. Rat FABP2 protein structure was retrieved in PDB database (http://www.rcsb.org/pdb). Coding region and structural domains were predicted with ORF finder.^5^ and Pfam,^6^ respectively. Identification of homologous regions between *P. olivaceus* FABP2 and other retrieved proteins, and the corresponding similarity was conducted using Clustal X (version 1.83)^7^ software. The amino acid sequences were compared to build a phylogenetic tree (NJ method, bootstrap 1000 times) using Phylip softwere (version 3.63).^8^ CondonW software was used to perform factorial correspondence analysis (FCA) for the nucleotide composition and codon usage of the 14 FABP2 genes. Genomic GC and GC3s contents for each species were obtained by searching the public database (http://www.kazusa.or.jp/codon/).

*P. olivaceus* FABP2 protein structure was modeled using Rat FABP2 as template on SWISS-MODEL server.^9^,^10^ The modeled structure was evaluated using PROCHECK, a protein structural quality analysis software.^11^ The Pymol program was used to overlap and compare the predicted FABP2 model with rat FABP2 structure.

## Results

### *P. olivaceus* FABP2 gene cloning and sequence analysis

Using zebrafish FABP2 mRNA as the initial query probes, four *P. olivaceus* FABP2 EST homologous sequences were found in the database. After removal of the terminal sequences, one full-length cDNA was assembled from the four EST fragments.

To validate the assembled cDNA sequence, a RT-PCR was conducted using primers flanking the ORF. When the extracted intestinal RNA sample was examined on a UV spectrometer, the readings were between 1.8–2.0 for OD260 /280, and >2.3 for OD260/230, which indicate that the RNA had high purity and could be used for RT-PCR procedure. The PCR product had a size of 700 bp, the subsequent cloning and sequence analysis confirmed that this fragment matched the assembled FABP2 cDNA sequence, which has validated *in silico* cloning result.

The predicted coding region of the cDNA is 399 bp in length and it contains an ATG start codon, and TAA stop codon. A stop codon TAA is located upstream of the same reading frame, the cloned sequence has thus proven to be a full-length gene. The longest deduced polypeptide has 132 aa.

The *P. olivaceus* FABP2 coding region has 52% GC, and 76.8% GC3s content, respectively. When compared at the genomic scale, the genomic GC content was circa 50% with no big difference among the 13 species including 9 mammals, zebrafish, *Branchiostoma belcheri tsingtaunese*, *X. laveis* and chicken. However, the average genomic GC3s content varied from 48.14% (African clawed frog) to 68.13% (Platypus). When only the FABP2 genes were considered, the genes from *P. olivaceus, D. rerio*, and *Bb. tsingtaunese* contained significantly higher GC (>50%) and GC3s (>60%) content than those from the other 11 vertebrate species (GC content <46%, and GC3s < 56%). Coincidently, such differences is correlated with the species evolutionary position, i.e. lower GC and GC3s content for the lower vertebrates FABP2 when compared to the higher animals. The comparison of codon usage of the sequences did not see apparent usage bias in the 14 species, as the frequency for each codon was statistically synonymous among the 14 homologous genes (p > 0.05).

The deduced *P. olivaceus* FABP2 polypeptide has an apparent molecular weight of 15.3 kD, with a theoretical isofocusing pH at 6.74. It has relatively higher content of Lys, Asp and Thr, each at 12%, 9% and 10% (Mol% total aa), respectively.

**Figure 1.**
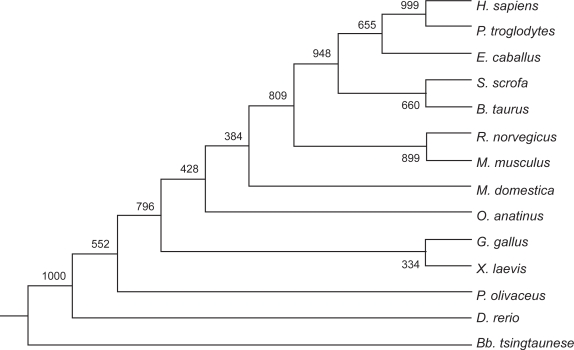
The phylogenetic tree of FABP2 nucleotides sequences. Abbreviations are the same as Table 1. The tree was constructed with the ClustalX and Phylip programs by the NJ methods.

In the molecular phylogenetic tree which was constructed using the FABP2 gene sequences from 14 animal species, *P. olivaceus* is closest to zebrafish by sharing the highest level of similarity (74.4%) in the coding region (Fig. 1). The similarity index is the lowest (42.8%) between *P. olivaceus* and *Bb. tsingtaunese* FABP2 genes. The similarity level with all the other species (Table 1) is between 60%–70%. When human and mouse FABP1 (liver type) were added to construct the phylogenetic tree, the FABP1 and the 14 intestinal FABP from this study were placed into two distinct clusters (data not shown). The liver type FABPs and intestinal type FABPs should be considered two paralogues belonging to one multiple gene family.

### Structural modeling and comparison of *P. olivaceus* FABP2

Using the Pfam program, a lipocalin signature domain was found in the *P. olivaceus* FABP2 protein. Lipocalin is a transportation structure for those typical hydrophobic small molecules such as lipid, cholesterol hormones, bile pigment, and Vitamin A.

**Figure 2.**
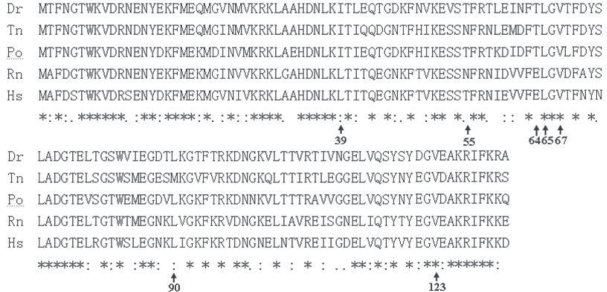
Amino acid sequences alignment of FABP2. Arrows point to the seven mutation sites of the FABP2s, “*”represents identical aa, “:”, and “.” are highly and relative highly conserved aa.

When the aa sequence from the deduced polypeptide sequences were aligned, it was found that *P. olivaceus* FABP2 had a very high similarity to the homologous proteins from human, rat, zebrafish, and puffer fish, with a similarity index at 65.1%, 67.4%, 75.7% and 75%, respectively (Fig. 2). In the 3D structure which was constructed using rat FABP2 as the template (PDB accession, 1ICM) with SWISS-MODEL program, 2 helices, 11 β sheets and 10 coils was found in the *P. olivaceus* protein model.

Further FABP2 structures for zebrafish, puffer fish, and human were all created using the same approach. These modeled proteins were compared (PROCHECK) for the structural parameters including aa ratio in the Ramachandran map, dihedral, covalent and overall. The results showed that aa ratio within the Ramachandran map was close to or exceeded 90%; the other three parameters were all statistically different (p < 0.5). Based on these data, the modeled protein structures should be considered stable enough for comparative analysis.

**Figure 3.**
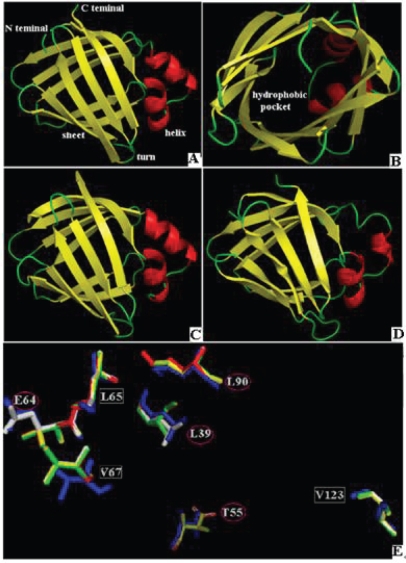
Comparison of the tertiary structures of FABP2s from *P. olivaceus (****A, B****)*, *R. norvegicus* (**C**), *H. sapiens* (**D**) and Spatial positioning of the seven aa in different FABP2 proteins from *P. olivaceus* (yellow), *D. rerio* (green), *T. nigroviridis* (red), *R. norvegicus (white)* and *H. sapiens (blue)* (**E**). The α-helices were highlighted in red, β sheets in yellow, coils in green. Amino acids in red circles are invariables; those in white boxes are variables.

*P. olivaceus* FABP2 protein tertiary structure comprises 10 anti-parallel β sheets, which twist and fold into a hydrophobic pocket. The yellow stripes are the β sheets, and the helices and coils are highlighted in red and green color, respectively (Fig. 3). These structures were all aligned to the corresponding sites predicted with Jpred. The *P. olivaceus* FABP2 was then over-laid with the homologous proteins from human and the other three species and each was marked with a different color. Using seven polymorphic sites in human FABP2 as the references, the corresponding positions (aa) in the other protein structures were highlighted, and all the rest were hidden. Four sites, L39, E64, T55 and L90, were found to have relatively bigger variation, and the other three sites, L65, V67 and V123, were very conserved among the five proteins (Figs. 2, 3E).

## Discussion

The FABP gene cloned from *P. olivaceus* in this study encodes for the intestinal type protein. It has a stop codon upstream of the start codon in the same translation frame, and the deduced polypeptide has a length of 132 aa. Both the DNA sequence and the deduced protein structure match the characteristics of the FABP protein family. Therefore, the cloned sequence has proven to contain the full-length coding region for the protein.

The cloned gene has very high homology to the intestinal FABP from other animal species. In the molecular phylogenetic tree, the deduced protein was placed together with other intestinal FABP retrieved from database, and separated from human and rat liver FABP which formed a separate cluster. It can be speculated that the intestinal FABP has begun to form a distinct type of protein since *Bb. tsingtaunese*. Intestinal FABP genes were studied quite extensively in zebrafish. This protein can be detected three days after fertilization. As the development of digestion system progresses, intestinal FABP gene will express regularly in specific sites. Consequently, the gene expression pattern was used as a marker for intestinal cell differentiation and development.^12^

During the evolutional history, an FABP family consisting of multiple types of proteins has formed. The encoding genes are located on different chromosomes. Searches in human genome database have found the muscle and heart type FABP genes on chromosome 1, liver type FABP gene on chromosome 2, intestinal FABP gene on chromosome 4, ileum FABP6 gene on chromosome 5, brain FABP gene on chromosome 6, skin and lipid cell FABP genes on chromosome 8 (http://www.ncbi.nlm.nih.gov/genome/guide/human/). Similarly, the intestinal type FABP gene is on chromosome 1, and the liver FABP on chromosome 7 in zebrafish. The chromosome 17 carries the brain FABP gene (http://www.ncbi.nlm.nih.gov/genome/guide/zebrafish/).

To identify the evolutionary pattern of the FABP2, this study compared the nucleotide composition, codon usage, and the protein structures of the homologous genes from 14 animal species. There is some variation in nucleotide composition across species and from lower organisms to poikilotherm animals; however, the percentage of each nucleotide remained the same. In higher vertebrates, the GC distribution is not synonymous as the functional genes are mostly located in the GC rich region. In addition, many species have shown codon bias toward G, or C tailing, which explains why codon usage bias is associated with differential distribution of the four nucleotides in the genome.^13^–^15^ The characteristics of codon usage are strongly correlated with the genomic GC content, especially the base composition at the third codon positions. 16–18 Results produced from previous studies on IAFBP have shown that the coding region is closely associated with GC and GC3s contents. Significant variation, especially GC3s, have been detected in different species, suggesting that this gene family has undergone selection pressure and corresponding adaptive nucleotide modification after long-term selection during the evolutionary history. However, no codon usage bias was found in the 14 FABP2 proteins (p > 0.05) in this study. This result suggests that that this gene may not be actively expressed in these species because highly expressed genes should have high bias for codon usage.^19^,^20^

Rat FABP2 crystal structure has been used as the standard reference for understanding the tertiary structure of the homologous proteins. This study used the SWISS-MODEL remote server to model IAFBP proteins from *P. olivaceus*, zebrafish, and puffer fish. The modeled protein structures basically overlapped with the FABP2 proteins from human and rat. Therefore, we can speculate that this gene family is largely conserved during the evolutionary process. In human FABP2 isoforms, the following aa (s) of L39, E64, T55, L90, L65, V67 and V123 all have some variations, which affect protein function. For instance, mononucleotide polymorphic mutation can change aa position 55 from Ala to Thr, which subsequently affects long-chain fatty acid hydrophobicity, fatty acid oxidation, and insulin resistance.

Mutations of L39G, E64G, L90G, L65G, V67G and V123G will affect protein stability. By comparing the same sites of the six FABP2 proteins from different species, four sites with alteration have been identified, while the other three sites were conserved. Relevant information can be used to further investigate the functional characteristics of the FABP2 proteins from different resources. In some human groups, the FABP2 was found to have position 54 aa changed from Ala to Thr due to mononucleotide polymorphism (SNP) and this change is associated with diabetes mellitus. The polymorphism of the promoter for this gene may control the mutation of Ala 54 into Thr, and the resultant changes in glycerol level and insulin sensitivity.^21^ The intron B of Human FABP2 gene contains the polymorphic trinucleotide (TTA) n repeats depending on individuals.^22^ Zebrafish FABP2 gene (accession: NC_007112:33448541-33451446) has 2906 bp in length, the nucleotide positions 1496,1514,1541, and 1561 all contain the microsatellite trinucleotide markers. Based on the analysis and observations, both FABP2 genes and proteins exhibit certain levels of polymorphism in human and zebrafish. The next step will be to analyze such phenomenon in *P. olivaceus,* in order to identify markers that can be used for molecular breeding to improve the quality of the fish muscle meat.

## Figures and Tables

**Table 1.** Data of FABP2 cDNA and genomic GC content of 14 species.

**Species**	**cDNA accession number**	**cDNA homology**	**GC content**	**FABP2 GC3s content**	**Genomic GC content**	**Genomic GC3s content**
Japanese flounder (*P. olivaceus*)	EU159579	100	52	76.8	51.41	60.18
Zebra fish (*D. rerio*)	NM_131431.1	74.4	49.5	64.3	50.42	56.43
Chicken (*Gallus gallus*)	NM_001007923	68.4	40.7	42.1	51.2	57.51
Opossum (*Monodelphis domestica*)	XM_001363273	67.6	44.9	55.7	47.41	53.76
Platypus (*Ornithorhynchus anatinus*)	XM_001512477	67.1	45.2	55.6	55.93	68.13
Rat (*Rattus norvegicus*)	NM_013068.1	65.6	42.9	46	52.6	60.48
Mouse (*Mus musculus*)	NM_007980	65.1	43.4	47.6	52.21	58.99
Chimpanzees (*Pan troglodytes*)	XM_001149448	65.1	38.1	32.3	54.78	63.63
African clawed frog (*Xenopus laevis*)	NM_001085877	64.9	40.7	38.6	46.93	48.14
Human (*H. sapiens*)	NM_000134.2	64.1	38.9	35.4	52.34	58.67
Cow (*Bos taurus*)	AY911349.1	64.1	38.9	34.1	53.56	62.48
Pig (*Sus scrofa*)	NM_001031780	63.1	37.9	36.5	54.48	64.93
Horse (*Equus caballus*)	NM_001081903	62.9	40.7	38.1	53.03	62.71
Lancelet (*Branchiostoma belcheri tsingtaunese*)	DQ531633.1	42.8	51	64.4	53.73	67.64
